# The prevention and treatment of breast cancer- related lymphedema: A review

**DOI:** 10.3389/fonc.2022.1062472

**Published:** 2022-12-06

**Authors:** Maureen P. McEvoy, Ameer Gomberawalla, Mark Smith, Francesco M. Boccardo, Dennis Holmes, Risal Djohan, Paul Thiruchelvam, Suzanne Klimberg, Jill Dietz, Sheldon Feldman

**Affiliations:** ^1^ Breast Surgery Division, Department of Surgery, Montefiore Medical Center, Montefiore Einstein Center for Cancer Care, Bronx, NY, United States; ^2^ Department of Surgery, Advocate Medical Group, Oak Lawn, IL, United States; ^3^ Department of Plastic Surgery, Northwell Health System, New Hyde Park, NY, United States; ^4^ Department of Surgery, San Martino Hospital, University of Genoa, Genova, Italy; ^5^ Department of Surgery, Los Angeles Center for Women’s Health, Los Angeles, CA, United States; ^6^ Department of Plastic Surgery, Cleveland Clinic, Cleveland, OH, United States; ^7^ Department of Breast Surgery, Imperial College, London, United Kingdom; ^8^ Department of Surgery, University of Texas Medical Branch(UTMB) Cancer Center, Galveston, TX, United States; ^9^ Department of Surgery, University Hospital Cleveland Medical Center, Cleveland, OH, United States

**Keywords:** breast cancer, lymphedema, breast cancer related lymphedema, axillary reverse mapping, LyMPHA, axillary surgery

## Abstract

**Background:**

Breast cancer- related lymphedema (BCRL) affects about 3 to 5 million patients worldwide, with about 20,000 per year in the United States. As breast cancer mortality is declining due to improved diagnostics and treatments, the long-term effects of treatment for BCRL need to be addressed.

**Methods:**

The American Society of Breast Surgeons Lymphatic Surgery Working Group conducted a large review of the literature in order to develop guidelines on BCRL prevention and treatment. This was a comprehensive but not systematic review of the literature. This was inclusive of recent randomized controlled trials, meta-analyses, and reviews evaluating the prevention and treatment of BCRL. There were 25 randomized clinical trials, 13 systemic reviews and meta-analyses, and 87 observational studies included.

**Results:**

The findings of our review are detailed in the paper, with each guideline being analyzed with the most recent data that the group found evidence of to suggest these recommendations.

**Conclusions:**

Prevention and treatment of BCRL involve a multidisciplinary team. Early detection, before clinically apparent, is crucial to prevent irreversible lymphedema. Awareness of risk factors and appropriate practice adjustments to reduce the risk aids are crucial to decrease the progression of lymphedema. The treatment can be costly, time- consuming, and not always effective, and therefore, the overall goal should be prevention.

## 1 Introduction

Breast cancer mortality is decreasing due to improved diagnostics and treatments. As survival is increased, the long-term sequelae of treatment and impaired quality of life become relevant. Breast cancer- related lymphedema (BCRL) affects about 3 to 5 million patients worldwide, with about 20,000 per year in the United States. BCRL rates range from 2% to 77% (1) based on the type of local–regional and systemic therapies.

BCRL results from disruption of the lymphatic system. This prevents adequate drainage, allowing lymph fluid to accumulate in the interstitial space (2–4). BCRL has been defined as a 2- cm increase in limb circumference, a 200- mm increase in limb volume, or a 5% to 10% change in limb volume compared to the unaffected arm (4–6).

Diagnosing pre-clinical lymphedema can be challenging and requires preoperative assessment and surveillance at standardized intervals (4, 7–10). There are several methods to detect and monitor lymphedema. There are no head-to-head comparison trials comparing all techniques (11). Increasingly, data and guidelines support the use of prospective surveillance programs to detect BCRL in its subclinical phase (12–15). Ideally, screening programs should use tools that are objective and reproducible.

Several risk factors have been identified for the development of BCRL. Risk factors are subdivided into patient-specific and treatment-specific risk factors. Patient- specific factors include body mass index (BMI) at the time of diagnosis, subclinical edema, and cellulitis on the side of treatment (4). The independent treatment- related risk factors for BCRL include lymph node surgery (16–20) and regional lymph node radiation (RLNR) (21–24).

Prevention of BCRL can be treatment specific. Decreasing the amount of axillary surgery in certain circumstances, mapping out the upper extremity lymphatics during surgery, and decreasing the amount of nodal radiation in appropriate cases can aid in the prevention.

The treatment of BCRL is non- surgical management and surgical management (25). The mainstay of non-operative treatment is complex decongestive therapy (CDT). Surgical management is another option, especially in patients who have no response to non-invasive treatment. The two surgical strategies are ablative and physiologic procedures.

Emerging evidence regarding biomarkers assesses the biological effects of physical exercise on breast cancer survivors (26). This allows for a more tailored rehabilitation plan based on the patient’s characteristics. Furthermore, evidence shows that surgical prehabilitation in women with breast cancer is feasible and well- received (27).

It is imperative to identify patients at high risk for developing BCRL so they can be monitored and treated before chronic, irreversible BCRL occurs. Early detection and treatment of subclinical BCRL can prevent progression to its chronic stage, thus eliminating morbidity and the need for more intensive, costly treatments (4, 12–15).

## 2 Methods

The American Society of Breast Surgeons (ASBrS) built a lymphatic surgery working group to develop guidelines on BCRL. During this process, the group proposed important factors regarding assessment, surveillance, risk factors, and prevention including decreased axillary surgery, decreased radiation to the axilla, and operative techniques to assist in the prevention and treatment including non-operative and operative. This review is a reflection of this process with articles selected based on the topics proposed as guidelines. While not a systemic review, we sought to identify high- quality research to support these guidelines. This was inclusive of recent randomized controlled trials, meta-analyses, and reviews evaluating the prevention and treatment of BCRL. There were 25 randomized clinical trials, 13 systemic reviews and meta-analyses, and 87 observational studies included.

## 3 Results

The findings of our review are detailed in the discussion of the paper. With each guideline being analyzed with the most recent data, the group found evidence to suggest these recommendations.

## 4 Discussion

### 4.1 Pathophysiology

BCRL results from disruption of the lymphatic system. This prevents adequate drainage that allows lymph fluid to accumulate in the interstitial space (2–4). First, it is important to differentiate between lymphedema and lymphatic insufficiency. Patients with lymphatic insufficiency seen by indocyanine green (ICG) lymphangiography may not have clinically appreciable lymphedema due to compensation by remaining lymphatics. However, these patients do have impaired lymphatic function and are therefore at risk for developing clinical lymphedema.

BCRL has been defined as a 2- cm increase in limb circumference, a 200- mm increase in limb volume, or a 5% to 10% change in limb volume as compared to the unaffected arm (4–6). The four stages of lymphedema based on the International Society of Lymphology are described as follows: Stage 0 is subclinical with no visible changes. Stage 1 is mild and soft edema pits, has no dermal fibrosis, and subsides with limb elevation. Stage 2 is moderate with loss of elasticity and evolution of dermal fibrosis and no decrease in swelling with arm elevation. Stage 3 is chronic and irreversible (4).

### 4.2 Assessment and surveillance

Diagnosing pre-clinical lymphedema can be challenging and requires preoperative assessment and surveillance at the standardized interval (4, 7–10).

There are several methods to detect and monitor lymphedema. There are no head-to-head comparison trials comparing all techniques (11). Recently, a randomized clinical trial compared bioimpedance spectroscopy (BIS) and tape measurement for BCRL surveillance. They found that BIS provides a more precise identification of patients likely to benefit from early compression intervention (28). The advantages and disadvantages of measures of BCRL are listed in **Table 1** (29).

**Table 1:** Assessment tools for BCRL.

Modality	Mechanism	Advantage	Disadvantage
Tape measure	Non- stretch tape measure at multiple points along the arm	Inexpensive and available	Time- consuming and lack reproducibility
Perometry	Frame of infrared light beam–receiver pairs to measure limb outline with sub-centimeter definition and thus derive limb volume by the disc model method	Quick, accurate, and highly reproducible	High up-front cost and large footprint for the equipment
Water displacement	Arm is placed in a cylinder of water, any displaced water is measured and compared to contralateral side	Accurate and inexpensive	Does not isolate the site of swelling, unhygienic, and time- consuming
Bioimpedance spectroscopy	Measures tissue resistance to the flow of electric current	Can detect subclinical lymphedema, high specificity, non-invasive	Expensive

BCRL, breast cancer-related lymphedema.

Circumferential sequential arm measurements are an acceptable assessment method. This involves a non-stretch tape measure at multiple points along the arm (30). This is an inexpensive and easily accessible method; however, it is time- consuming and can be inconsistent with various users (31), leading to inaccurate measurements. Specificity can be as low as 73% (32). It also detects a late stage of lymphedema (4, 33, 34).

Perometry is a measurement option utilizing infrared light to measure limb volume (35). The patient places each arm into a large frame that sends infrared light beams inward while a computer calculates limb volume using a disc model. The benefits include being quick, accurate, and highly reproducible; however, it comes at a high up-front cost and a large footprint for the equipment (36). Compared to a tape measure, it has a smaller standard error with a narrower confidence interval (4, 37).

The water displacement technique is accurate and inexpensive. It is performed by placing each arm in a large cylinder of water and comparing the displaced water to the other arm. Disadvantages include that it does not isolate the site of swelling, is time- consuming and unhygienic, and requires a strict protocol (4, 31).

BIS can detect subclinical lymphedema using a non-invasive tool that measures tissue resistance to the flow of electric current to determine the quantity of extracellular fluid. A scanning device passes a small, painless electrical current through the limb and measures resistance to the current. Measurements are taken at multiple locations on the arm. The specificity is 80%–99% (12, 38–40). Advantages include fast detection and limited need for staff and space (38, 41–43). Limitations are the expense, approximately $5,000 for the equipment and $12,000 annually for data storage. The data for BIS have been encouraging. Soran et al. showed that monitoring with BIS reduces the incidence of subclinical BCRL from 36.4% to 4.4% (12). Similarly, Whitworth et al. demonstrated that 3% of patients had unresolved clinically significant BCRL when BIS was used as surveillance, which is lower than reported in contemporary studies (4, 44, 45).

Subjective measures for BCRL include patient- reported outcomes (PROs). Swelling, pain, heaviness, aching, numbness, stiffness, and impaired arm mobility are the most common PROs (46–49). Data suggest that PROs should be evaluated at intervals for 2–6 years after treatment (4, 23, 46).

Increasingly, data and guidelines support the use of prospective surveillance programs to detect BCRL in its subclinical phase (12–15). Ideally, screening programs should use tools that are objective and reproducible. An initial preoperative measurement should be obtained followed by surveillance, including regularly scheduled postoperative measurements, every 3 months for the first 2 years and then every 6 months for 3–5 years for all patients undergoing axillary surgery (4, 29).

### 4.3 Reduction of risk

Several risk factors have been identified for the development of BCRL. Risk factors are subdivided into patient-specific and treatment-specific risk factors. Patient- specific factors include BMI at the time of diagnosis, subclinical edema, and cellulitis on the side of treatment (4). The independent treatment- related risk factors for BCRL include lymph node surgery (16–20) and RLNR (21–24) (**Table 2**).

**Table 2:** Risk reduction.

Patient- specific factors	Treatment- specific factors
BMI	Axillary surgery
Subclinical edema	Nodal radiation
Cellulitis	

BMI, body mass index.

### 4.4 Patient- specific factors

BMI has been widely reported to be associated with BCRL (50–52). The risk of lymphatic dysfunction increases with elevated BMI and is almost universal once BMI exceeds 60. Individuals with a BMI greater than 30 kg/m^2^ are more likely to develop secondary upper extremity lymphedema after breast cancer treatment (52, 53). Obesity has a negative impact on lymphatic density in subcutaneous tissue, lymphatic endothelial cell proliferation, lymphatic leakiness, collecting-vessel pumping capacity, and clearance of macromolecules (54). Helyer et al. (52) compared patients with a BMI greater than 30 kg/m^2^ to patients with BMI less than 25 kg/m^2^, finding an odds ratio of 2.93 of developing BCRL in the higher BMI cohort (4).

Subclinical edema is a risk factor for BCRL. Specht et al. reported on the correlation between subclinical swelling and BCRL. They found that early on, both small and large volume increases were associated with increased BCRL (55). Studies like these emphasize the utility of early screening for limb volume increases (22, 24).

Cellulitis is a known risk factor for lymphedema. A large prospective cohort study by Ferguson et al. conducted a large prospective study proving this fact, showing a mean difference in arm volume by about 3% in those with cellulitis (18, 19, 56).

Avoidance of ipsilateral venipuncture, blood pressure readings, and air travel has been considered imperative in BCRL prevention (4). This basis is anecdotal and derived from a small number of patients (57, 58). Many studies have actually demonstrated that these practices are not inclined to greatly influence the development of lymphedema (4, 19, 22, 59). Ferguson et al. reported on over 3,000 patients who were screened prospectively for lymphedema using a perometer. At each measurement, patients reported the number of blood draws, injections, blood pressure measurements, trauma, and flights taken. There was no significant association between volume change and any of these variables among this cohort. It has been reported that flying increases the development of lymphedema due to changes in cabin air pressure. However, it is unclear if the reported lymphedema was preexisting or influenced by other factors in these studies (60). Kilbreath et al. evaluated changes in arm volume after short- and long- distance flights and found no change in arm volume as measured by BIS (61). These practices can burden patients with added anxiety and avoidance strategies (19, 22, 59).

However, while avoidance of these activities may not mitigate the development of lymphatic insufficiency, they may avoid hastening the onset or exacerbation of clinically evident lymphedema. The National Lymphedema Network position paper advises the following for risk reduction: if required to have venipuncture, ask to use a non-lymphedema limb if possible. Use an uninvolved or not at-risk extremity when taking blood pressure, and request a hand blood pressure measurement if possible. Patients can make an informed personal decision regarding compression garments during air travel; regardless, it is important to move around, exercise the at-risk body part, and maintain good hydration during air travel (62). Therefore, patients should not be overly cautioned against these things if they are necessary for medical (IV and injections) or personal (travel) reasons but should try to avoid using the affected extremity if not necessary.

### 4.5 Oncologic treatment- specific factors

At this time, there are inconclusive results identifying a direct correlation between taxane- based chemotherapy and BCRL. Taxane- based chemotherapy is the conventional treatment for breast cancer and can significantly improve progression-free survival and overall survival of patients (63, 64). Taxane- based chemotherapy can cause fluid retention in extremities. There has been some correlation discussed in the literature; however, it is inconsistent. Swaroop believed that taxane- based chemotherapy did not increase the risk of BCRL (65), whereas Cariati et al. showed there was a correlation (66).

### 4.6 De-escalating axillary surgery

Axillary nodal status remains among the most important variables for both prognosis and adjuvant therapy when treating breast cancer (67–69). With the increased utilization of sentinel lymph node biopsy (SLNB) and data supporting the de-escalation of axillary surgery, axillary lymph node dissection (ALND) has been avoided more commonly (**Table 3**). The data from the American College of Surgeons Oncology Group (ACOSOG) Z0011 and AMAROS have driven this trend (70, 71). These two studies demonstrate that ALND can be avoided in certain cases. ACOSOG Z0011 randomized patients with clinically node- negative disease who underwent a lumpectomy and had two or fewer positive sentinel nodes to undergo completion axillary dissection versus no further surgery. At 10 years, the overall survival, disease- free survival, and local recurrence rates were equivalent (70). AMAROS randomized patients with T1–T2 patients undergoing lumpectomy or mastectomy, with clinically negative nodes, to receive completion dissection or axillary radiotherapy in the case of a positive sentinel node and found comparable local control, with less morbidity in the radiotherapy group (71). This is likely due to the fact that during surgery, lymph nodes are removed including the lymphatics, and this removal has a greater increase in lymphedema than leaving the anatomy intact and providing radiation.

**Table 3:** De-escalation of axillary surgery.

Considerations	Evidence
Avoid ALND when possible	ACOSOG Z0011 AMAROS
Avoid SLNB in select patients	CALB 9343, SentiNot
SLNB after neoadjuvant chemo	ACOSOG Z1071
Targeted axillary dissection after NAC	Caudle et al.
Axillary reverse mapping	Klimberg et al.
LyMPHA procedure Avoid SLNB in prophylactic mastectomy	Boccardo et al.

ALND, axillary lymph node dissection; ACOSOG, American College of Surgeons Oncology Group; SLNB, sentinel lymph node biopsy; NAC, neoadjuvant chemotherapy; LyMPHA, Lymphatic Microsurgical Preventive Healing Approach.

There has not been conclusive evidence to define the number of retrieved lymph nodes to correlate with lymphedema. Vicini et al. (43) demonstrated a trend of increased lymphedema when four or more lymph nodes were retrieved; however, this was not statistically significant. Engel et al. (72) demonstrated that retrieval of 10 or more lymph nodes was significantly associated with lymphedema. However, there is no consensus regarding the number and lymphedema. In a recent study of 936 patients, there was no association between the number of nodes retrieved and lymphedema (73).

These findings have motivated the consideration of eliminating unnecessary lymph node analysis in certain circumstances. As part of Choosing Wisely Campaign, which encourages doctors and patients to question commonly used tests and treatments, the ASBrS recommended against the use of routine ALND in patients undergoing lumpectomy (74). In addition, the Choosing Wisely guidelines and the Society of Surgical Oncology recommended against routine use of SLNB in women over 70 with hormone receptor- positive HER2- negative disease. Hughes et al. showed that the risk of axillary recurrence was low among women who did not undergo axillary surgery. Furthermore, there was no difference in breast cancer- specific mortality in those who underwent axillary surgery and those without axillary surgery in women over the age of 70 (75, 76).

Patients undergoing prophylactic mastectomy, with proper imaging, such as MRI, do not need sentinel node biopsy.

To aid in this avoidance, the SentiNot study evaluated injection with superparamagnetic iron oxide nanoparticles for patients with ductal carcinoma *in situ* (DCIS) to minimize unnecessary SLNB. Patients who were found to have the invasive disease could go on to have SLNB even after a mastectomy, as this technique allows SLNB to be performed several weeks after injection. They found that 78.3% of patients with DCIS avoided SLNB (77).

Node- positive patients who have shown a good clinical response after neoadjuvant chemotherapy can avoid ALND in certain circumstances. This has been shown to be oncologically safe. The ACOSOG Z1071 (78) tested if SLNB could accurately assess nodal response after neoadjuvant chemotherapy (NAC) (4). They evaluated over 600 patients who were initially node- positive and received chemotherapy, followed by both SLNB and ALND. They found the false- negative rate (FNR) to be 12.6% by identifying cancer in nodes other than the sentinel node. The use of a dual tracer increased the likelihood of finding a node and lowered the FNR. In addition, the FNR improved as the number of removed SLNs increased: 31%, 21%, and 9.1% when one node, two nodes, or three nodes were removed, respectively (4). The sentinel node biopsy following neoadjuvant chemotherapy (SN FNAC) (79) and GANEA2 (80) study found similar results.

Caudle et al. (81, 82) localized a clipped node with iodine-125 radioactive seed to increase the accuracy of SLNB after neoadjuvant therapy. This technique decreased the false- negative rate, with the lowest FNR reported at 2%. Of note, 23% of clipped nodes were not the sentinel node, highlighting the importance of localization of the clipped node. Of note, these patients receive radiation therapy (XRT). This technique can also be performed using an injection of carbon particles (83), or ICG, and the use of other localization devices (84). Simons et al. reported on a prospective registry trial using magnetic seeds to locate clipped nodes and had 100% seed placement and removal (85). Miller et al. found similar success with magnetic seed for lymph node localization (86).

With reliable SLNB after NAC, ALND can be avoided in patients with a good clinical response.

### 4.7 Surgical prevention

#### 4.7.1 Axillary reverse mapping

Axillary reverse mapping (ARM) is a technique used to distinguish the lymphatic drainage of the arm from that of the breast. It identifies upper extremity lymphatics coursing through the axilla to minimize unnecessary disruption of the arm lymphatics while performing lymph node surgery (87–89). ARM maps the (UE) lymphatics with blue dye while using technetium-99 in the breast, allowing for differentiation of lymphatics draining the breast (hot) and the UE (blue) (4). Variations in UE lymphatic drainage patterns exist and may coalesce or cross over with those draining the breast.

There are three factors to consider with ARM: feasibility, prevention of BCRL, and oncologic safety. A large phase 2, single- arm trial with 654 patients evaluated the feasibility of ARM. They reported the identification of blue UE lymphatics in 29% of SLNB patients and 72% of ALND patients (90–98). Boneti et al. identified blue in 39% of those patients having SLNB and in 81% of patients having ALND (92). Wijaya et al. performed a review and meta-analysis of 29 studies with 4,954 patients combined. They found the feasibility to be 37% in SLNB and 82% in ALND (99). The larger percentage of identification in ALND is due to the larger operative field and a greater area of dissection, aiding in visibility.

In terms of the prevention of BCRL, Tummel et al. prospectively reported on patients in a single study. They found that the rate of BCRL after SLNB with ARM was 0.8% and ALND with ARM was 6.5% with 26 months follow- up (98). In the meta-analysis by Wijaya et al., they found that the BCRL rate was 2% in those with SLNB and ARM and 14% with ALND and ARM (99).

In an analysis of five randomized controlled trials evaluating ALND versus ALND with ARM, all five studies favor ALND + ARM for lower BCRL rates (100).

In a small fraction of patients, the ARM (blue) node will also be the sentinel lymph node (SLN). This can call into question oncologic safety. Tummel et al. (93) found that crossover SLN (hot) and blue were seen in 3.8% of SLNB procedures. Crossover happened in 5.6% of those undergoing ALND. In the subset of patients in which an identified blue lymphatic was transected, there was an overall lymphedema rate of 18.7% when not re-anastomosed compared to 0% when re-anastomosed at 14 months of follow-up (98). Of note, the axillary recurrence rate was 0.2% in those with SLNB and 1.4% in those with ALND.

Yuan et al. described the identification and Preservation of the Arm lymphatic system (DEPART) technique, which uses ICG injected intradermally to map out the course of the arm lymphatics. They identified arm nodes using methylene blue and then injected the node with ICG to flow along several lymphatic channels. They randomized patients to ALND alone versus ALND with DEPART. They found arm nodes in 83.2% of patients and subsequent nodes in 558 (97.4%) patients. The lymphedema rate was 3.3% in the study group *vs.* 15.3% in the ALND alone group (101).

Future work includes the Alliance trial A221702, a randomized prospective phase III trial that studies how well axillary reverse mapping works in preventing lymphedema in patients with breast cancer undergoing axillary lymph node dissection. Patients will be randomized to ARM versus no ARM. This randomized trial will help establish the change in practice as needed based on the evidence found.

#### 4.7.2 Lymphatic microsurgical preventive healing approach

A logical progression from ARM is the Lymphatic Microsurgical Preventive Healing Approach (LyMPHA) procedure. LyMPHA provides a preventative lymphovenous anastomosis. During an axillary node dissection, if the malignant node is draining the arm, a lympha-vascular anastomosis can be performed (4). In a randomized study in 2011, Boccardo et al. compared LyMPHA in 46 patients undergoing complete ALND. At 6 months of follow-up, they showed that one (4.34%) patient with LyMPHA developed lymphedema versus seven (30.43%) control patients (102). Four- year follow- up demonstrated minimal lymphedema, with only 3/74 (4%) having BCRL (103). Feldman et al. reported a lymphedema rate in three of 24 (12.5%) undergoing LyMPHA compared to four of eight (50%) who did not have the procedure. They concluded that LyMPHA is feasible, safe, and effective for the primary prevention of BCRL (4, 104).

Simplified Lymphatic Microsurgical Preventing Healing Approach (S-LyMPHA) is a simplified modification of LyMPHA. During the ALND, transected blue lymphatic channels are identified and invaginated into a cut end of a vein and secured in place by suture. The microscope is not needed and can be performed by the operating surgeon. Of 380 patients who underwent ALND, there was a significantly lower rate of BCRL (105).

Johnson et al. recently described the benefits of LyMPHA in those patients undergoing ALND and nodal radiation. They performed a literature review and included 19 studies. The pooled cumulative incidence of lymphedema was 33.4% in those undergoing ALND and RLNR versus 10.3% in those undergoing ALND, RLNR, and LyMPHA (*p* = 0.004) (106).

ARM and LyMPHA help prevent BCRL by identifying nodes draining the arm, as well as establishing a reconnection for the disrupted lymphatics. Further randomized multicenter trials would need to be conducted to establish more long-term data.

#### 4.7.3 Minimize radiation to prevent lymphedema

For patients who undergo lumpectomy, radiation therapy is usually recommended. Traditionally whole breast radiation is recommended; however, there are data that show that in patients with early- stage, favorable breast cancer, partial breast irradiation (PBI) is safe and effective (4). PBI can be delivered as an external beam radiation therapy (EBRT), as targeted intraoperative radiation (TARGIT-IORT), as a balloon catheter, or as interstitial catheter brachytherapy. There have been two large prospective randomized trials comparing whole breast radiation to both forms of IORT, electron and 50-kV photons, showing low local recurrence rates and excellent overall survival outcomes (107, 108).

Vaidya et al. (109) randomized patients to an external beam of whole breast radiotherapy or TARGIT-IORT, a one-time dose immediately after lumpectomy in the operating room. They have found no statistical difference for local recurrence-free survival, mastectomy-free survival, distant disease-free survival, overall survival, and breast cancer mortality. They found that patients getting IORT compared to EBRT reported less general pain and arm symptoms as well as better overall functioning. The TARGIT-A trial has not yet reported specifically on breast cancer- related lymphedema. We hypothesize that since RLNR is avoided with TARGIT-IORT, the BCRL rate will likely be lower compared with that of EBRT. Interestingly, a review of the radiation fields used in the ACOSOG Z0011 trial found that in approximately half the patients, the radiation fields were within 2 cm of the humeral head, likely causing substantial incidental axillary irradiation (4, 110). Lymphedema rates from TARGIT-A would be valuable data to be able to identify ways to prevent lymphedema with more directed radiotherapy.

Obi et al. (111) reported outcomes and acute toxicities from a single institution with intraoperative radiation. They found the rate of arm lymphedema was 0.5% (1/201) with a median follow- up of 23 months.

Warren et al. (24) conducted a prospective cohort study of 1,476 breast cancer patients using volume measurements with a perometer to assess the impact of various radiation techniques on rates of BCRL. They found that the 2-year cumulative incidence of BCRL was 6.8% at 25.4 months. The cumulative incidence defined by the type of radiation was as follows: 3.0% no radiation, 3.1% breast or chest wall alone, 21.9% supraclavicular (SC), and 21.1% with SC and posterior axillary boost. Interestingly, they treated 6% of patients with PBI. In a univariate analysis for factors with risk of lymphedema, those who underwent PBI only had a hazard ratio of 0.38; however, this was not statistically significant due to the low numbers of patients. This demonstrates the potential benefit of PBI in avoiding RLNR and thus decreasing the risk of BCRL (4). This has been suggested in other studies as well (112).

The omission of radiation has come into acceptance by older patients. CALGB 9343 randomized women older than 70 years with T1N0M0 ER-positive breast cancer who received lumpectomy and tamoxifen to radiation or no radiation. Among these two cohorts, there were no differences in cancer-specific survival, overall survival, or breast preservation rates. Not surprisingly, there was a statistical difference in locoregional control 98% versus 90% in the tamoxifen- only group (75). They concluded that women over the age of 70 with favorable, small estrogen- responsive tumors could avoid radiation if they commit to taking endocrine therapy (4).

By finding ways to minimize and possibly avoid radiation, BCRL rates can be improved.

### 4.8 Lymphedema treatment

#### 4.8.1 Non-surgical management of arm lymphedema

For patients in which lymphedema has been established, there are both non-operative and operative management options (**Table 4**). The mainstay of non-operative treatment is CDT. This is the universal first -line therapy. There are two phases consisting of reduction and maintenance. The reduction phase includes manual lymph drainage, sequential gradient pump, exercises, low-stretch bandages, and skin care. The maintenance phase consists of compression garments, exercises, and skin care.

**Table 4:** Treatment of lymphedema.

Non-surgical treatment	Surgical treatment	
Complex decongestive therapy	Ablative	Physiologic
Reduction phase	Liposuction/debulking	Lymphaticolymphatic bypass
Manual phase		Vascularized lymph node transfer
		Lymphovenous anastomosis

There has not been one strategy identified as the most beneficial. A randomized control trial compared complex decongestive therapy over standard compressive therapy reporting no significant difference in the percent volume reduction of the arm at up to 52 weeks. In addition, the PROs of quality of life (QoL) did not differ (4, 113). Compression garments have been shown to prevent progression in subclinical BCRL as well as reduce arm volume (15, 114, 115). Manual lymphatic drainage is important for volume reduction. It has been shown to be beneficial to patients with mild-to- moderate BCRL in conjunction with compression bandaging (116).

Physical therapy by lymphedema- trained therapists has been shown to be more beneficial than just patient education and physical therapy alone (14, 117). Exercise with aerobic and resistance exercises does not incite or exacerbate BCRL (118–122). The data suggest that monitored management with trained physical therapists is better than self-directed treatment (123).

Early detection and intervention are integral parts of preventing the progression of BCRL. A randomized trial from Madrid enrolled 120 patients who underwent ALND and had no evidence of BCRL. Patients were randomized to a program of manual lymphatic drainage, massage, and exercise or BCRL education alone. At 1 year follow- up, 7% of patients in the intervention arm developed BCRL, compared to 25% in the education- only group (14). An additional study randomized 65 women who underwent ALND to prospective monitoring and treatment with physiotherapy versus surveillance. At 2 years, the incidence of BCRL was 11% in the early intervention group compared to 30% in surveillance alone (117). This data support the recommendation that early detection and monitored treatment are beneficial in preventing progression (4).

#### 4.8.2 Surgical treatment of arm lymphedema

Surgical management is another option, especially in patients who have no response to non-invasive treatment. The two surgical strategies are ablative and physiologic procedures.

Ablative procedures remove edematous tissue, thus reducing limb volume. Liposuction or suction-assisted protein lipectomy (SAPL) is preferred over debulking procedures that require skin grafting. Liposuction/SAPL has been shown to have significant volume reductions (124–126). However, compression garments must be worn continuously to maintain the decreased volume (125, 126).

Physiologic procedures work by rebuilding and directing axillary lymphatic drainage. This is performed with re-anastomosis of lymphatics to distal tissue or veins.

Procedures utilizing distal tissues generally involve lymphatic grafts or vascularized flaps containing lymphatic soft tissue. The lymphaticolymphatic bypass procedure involves an anastomosis between healthy lymphatic tissue of the lower extremity to the affected arm’s axillary lymphatics and supraclavicular lymphatics. This has been shown to produce long-term patency and reduce upper extremity volume (127, 128).

Another major surgical treatment involves vascularized lymph node transfer (VLNT). A free flap of lymph node tissue is harvested with its vascular supply from a donor site and introduced to the affected limb. Blood supply is anastomosed from the lymph node flap’s blood vessels to the native axillary blood vessels (129, 130). There has been a reduction in limb volume following this procedure, circumference differentiation of 7.3%, and a reduction rate of 40.4% at a mean follow- up of 39.1 months. Furthermore, by mapping the lymphatic drainage of the donor site, selective removal of lymph nodes can be achieved to decrease the risk of lymphedema in the donor site (131).

A combination of SAL and VLNT has been shown to have a statistically significant reduction in arm circumference (132). Emerging evidence regarding microvascular breast reconstruction and lymph node transfer for post- mastectomy patients has been shown to relieve the effects of lymphedema after surgery (133).

Lastly, lymphatic venous anastomosis (LVA) uses proximal tissue instead of grafts to reestablish lymphatic drainage. Multiple lymphatic vessels are anastomosed to venules, allowing lymphatic drainage into the venous system. Chang et al. performed 89 LVAs in women with upper extremity lymphedema. Symptom improvement was reported in 96% of patients, and the mean volume reduction was 42% overall (134, 135).

There are no randomized clinical trials yet to fully compare and evaluate the benefits of these surgical procedures.

### 4.9 Study limitations

This study does not provide a systemic review of the literature regarding the prevention and treatment of BCRL. It provides a comprehensive review, giving evidence for guidelines being developed by the ASBrS. The bias is related to the expert panel selecting the topics to generate the guidelines.

## 5 Conclusion

BCRL is a challenging consequence of breast cancer treatment. Various methods of surveillance, early detection, avoidance of practices, and treatment adjustments can improve the outcomes.

## Author contributions

MM: substantial contributions to acquisition of data and involved in drafting and revision of the manuscript. AG, MS and FB: substantial contributions in revising the manuscript. DH, RD, PT, SK and JD: equal contributions to acquisition of data and in the revision of the manuscript. SF: substantial contributions to data acquisition, involved in drafting and revising the manuscript. All authors contributed to the article and approved the submitted version.
